# Locum doctor working: A qualitative exploration of the implications for learning and professional development

**DOI:** 10.1111/medu.70099

**Published:** 2025-11-23

**Authors:** Jane Ferguson, Gemma Stringer, Kieran Walshe, Thomas Allen, Christos Grigoroglou, Evangelos Kontopantelis, Darren M. Ashcroft

**Affiliations:** ^1^ Health Services Management Centre University of Birmingham Birmingham UK; ^2^ Alliance Manchester Business School University of Manchester Manchester UK; ^3^ Manchester Centre for Health Economics, Division of Population Health, Health Services Research and Primary Care University of Manchester Manchester UK; ^4^ Danish Centre for Health Economics University of Southern Denmark Denmark; ^5^ NIHR School for Primary Care Research Centre for Primary Care, Division of Population Health, Health Services Research and Primary Care, University of Manchester Manchester UK; ^6^ Division of Informatics, Imaging and Data Science, University of Manchester, Manchester, UK g NIHR School for Primary Care Research, Centre for Primary Care University of Manchester Manchester UK; ^7^ NIHR Greater Manchester Patient Safety Research Collaboration University of Manchester Manchester UK; ^8^ Centre for Pharmacoepidemiology and Drug Safety, School of Health Sciences, Faculty of Biology, Medicine and Health University of Manchester Manchester UK

## Abstract

**Background:**

In the English National Health Service, and other health care systems internationally, there have been growing numbers of doctors working on a short‐ or long‐term temporary basis as ‘locums’. Social environments and professional relationships are fundamental to learning in clinical contexts; however, locums are often positioned at the periphery of the organisation and the clinical team. An examination of locum learning and continuing professional development is vital to understanding the implications of temporary working for the growing numbers of mobile doctors, often working at the margins of the medical workforce, and whose career trajectories may diverge from traditional models.

**Methods:**

Qualitative interview and focus group data were collected from 130 participants, including 88 professionals and 42 patients, between March 2021 and April 2022 in primary and secondary health care organisations in the English NHS. Participants included locums, patients, permanently employed doctors, nurses and other health care professionals with governance and recruitment responsibilities for locums. Data were analysed using reflexive thematic analysis and abductive analysis.

**Results:**

Four themes were developed from the data: (1) exclusion from formal and informal learning opportunities; (2) self‐directed learning and workarounds; (3) decline in knowledge and clinical skills; (4) effects on the professional development of the wider team. Locums were frequently excluded from feedback and learning opportunities because they were considered expensive and not the responsibility of the organisation and there to work, not to train. This meant that professional development was often the responsibility of the locum, self‐directed and divorced from context. Locums often did not take on educational supervision roles for the wider team, meaning wider learning and development were disrupted or paused.

**Conclusion:**

To address the challenges locum working might bring for learning and professional development, professional bodies should provide guidance for locum doctors highlighting the risks associated with taking on locum work before medical knowledge and experience are established. To improve quality and safety, organisational leaders should include locums in developmental opportunities. Finally, policy makers need to strike a balance between using locums to address short‐term workforce quotas and the long‐term impact on the knowledge and development of the workforce and patient safety.

## Abstract 2

Does locum working hinder or enhance doctors' learning and professional development? Our latest study explores how locum roles shape opportunities for growth, experience, and career development. #MedEd #Workforce

## 1 INTRODUCTION

Lifelong learning in medicine is a fundamental principle that enables doctors to remain up‐to‐date and fit to practice throughout their careers.^1^ Once qualified, doctors learn continuously through a blend of both formal and informal education involving, for example, accredited courses, experiential learning, observation, role modelling, patient and peer feedback and mentoring and supervision.^2^ In the English National Health Service (NHS), formal continuing professional development (CPD) is a principal component and requirement of the clinical governance quality framework known as revalidation.^3^ All registered doctors in the United Kingdom are required to demonstrate they are up‐to‐date and fit to practice. This is achieved by providing evidence of their professional development at annual appraisals and for a five‐yearly formal revalidation recommendation from their employer to the General Medical Council.^4^ While all qualified doctors are professionally obliged to take part in formal CPD activities to keep up‐to‐date with best practices and address deficits in knowledge and practice post qualification,^5^ the majority of learning occurs through informal learning, such as day‐to‐day clinical practice, reflection and collaboration.^6^, ^7^

Social environments and professional relationships for learning in clinical contexts matter.^8^ Evidence suggests that medical education is a socially driven,^9^ actively negotiated process involving professional reproduction where medical knowledge is learned from more senior colleagues^10^ and identities are developed through interactions.^11^, ^12^ Medical education has been described as an ‘identity development process’ where learning occurs through identifying and resolving problems^2^ and newly qualified doctors ‘consolidate their acquisition of professional attitudes and values and crystallize their professional identity’.^13^ However, recently, there have been significant shifts in doctor career trajectories with a growing proportion of newly qualified doctors choosing not to go straight into specialty training and instead opting for ‘non‐training’ positions, such as working on a temporary basis as a ‘locum’.^14^, ^15^ These changes have had implications for collegiality, professional identity, group relations and team functioning^16^ and, therefore, potentially learning and professional development.

Internationally, employment relationships between doctors and organisations have evolved in recent years with more doctors working as locums.^17^, ^18^, ^19^, ^20^, ^21^ Locum work varies from the very short term (a single shift) to longer term (weeks, months or even sometimes years). All doctors in the United Kingdom, other than qualified doctors in their first year of training, can work as a locum. In the United Kingdom, locums are a heterogeneous group with different contractual arrangements and working patterns; some locums work exclusively as locums and take on only temporary positions, while others have substantive positions in a health care organisation, but also do some locum work.^19^ Locum pay scales can be significantly higher than permanent doctor rates, and NHS spending on temporary staff, including locums, increased between 2021 and 2022 from £3.45 billion to £5.2 billion.^22^, ^23^

In the NHS, most doctors are directly employed by NHS organisations with access to organisationally funded formal CPD and informal educational opportunities. While postgraduate learning represents the longest portion of a doctor's learning journey, evidence suggests that the ways doctors engage in lifelong learning may not be aligned with formal CPD processes and practices, which have been criticised as being ineffective, impractical and decontextualised.^24^ Locum working presents additional challenges to professional development because doctors are working in a peripatetic and episodic way, meaning access to developmental opportunities can be limited, fragmented or prioritised for permanent employees.^25^ Evidence suggests that locum doctors can face challenges accessing and evidencing the CPD required by the regulator for the purposes of revalidation.^26^, ^27^ While recent research suggests there is value in episodic professional relationships, including variety and diversity in feedback,^28^ longer term, continuous relationships have been found to produce better educational and patient outcomes than shorter‐term, episodic relationships.^29^, ^30^, ^31^

Despite increases in locum working across all stages of doctors' careers, the learning and professional development of locums remain under‐researched. Existing research has focussed on workforce issues,^32^ patient safety^25^ and regulatory oversight,^26^ with limited focus on how locums learn throughout their careers, particularly in peripheral contexts. By exploring locum learning across all career stages, this research addresses a critical gap in understanding professional development in non‐standard contexts and provides vital information to inform educational policy, workforce planning and organisational approaches. It also informs wider debates relating to equity of access to medical education in what are often a marginalised cohort of the medical workforce.

## 2 METHODS

### 2.1 Study design and setting

We conducted qualitative interviews with 88 locums and people who work with locums, and 42 patients took part in either a focus group or a one‐to‐one interview. Using purposive, snowball and convenience sampling, participants working in health care were recruited through 11 organisations including NHS trusts, primary care practices, statutory NHS bodies and locum agencies. Patients were recruited through patient and contributor forums. Organisations and patient forums were identified through stakeholder intelligence, including our project advisory group. Participant demographics were closely monitored to ensure diversity across a range of roles and representation in terms of gender and ethnicity (see Tables 1 and 2).

**TABLE 1:** Participant characteristics.

Characteristic		Professionals (n = 88)	Patients (n = 42)
Mean age Professionals (n = 81) Patients (n = 38)		46	59
Gender: women, men Professional (n = 88) Patients (n = 42)		49, 39	24, 18
Ethnicity Professionals (n = 80) Patients (n = 38)	White English	45	30
	Any other White background	12	4
	Mixed/multiple ethic group	3	1
	Asian/Asian British	15	2
	Any other Asian background	2	0
	Black/African/Caribbean/Black British	2	1
	Other ethnic group	1	0

**TABLE 2:** Health care organisations and participant roles.

Health care organisation type	Participant roles	Total
Primary care	GP × 6 Managing director × 1 Non‐clinical partner × 1 Nurse × 1 Practice manager × 4	13
Secondary care	Assistant psychologist × 1 Clinical director × 1 Clinical lead × 1 Clinical pharmacist × 1 Consultant × 9 Deputy medical director × 2 Director of patient experience × 1 Medical director × 2 Medical staffing × 6 Nurse × 3 Responsible officer × 3 Senior manager × 3	35
Primary care	Locum	19
Secondary care	Locum	12
Primary and secondary care	Locum	2
Locum agencies	Locum agency responsible officer × 4 Director × 1	5
Other	Advisor to NHS × 2	2
Total		88

### 2.2 Data collection

Three semi‐structured interview and focus group guides were developed for locums, people working with locums and patients. Schedules were developed using themes identified in our earlier narrative literature review and qualitative work that explored locum working and quality and safety.^16^, ^33^ Schedules were designed with the overall aim of exploring how locum doctor working may affect quality and safety, and participants were encouraged to discuss any issues they felt were relevant to locum work. Schedules used with locums and people who worked with locums (including those with governance and recruitment responsibilities for locums) contained questions specific to professional development, involvement in multi‐disciplinary team meetings and learning and educational activities. We also asked about why locums were used, reasons doctors chose locum work, issues relating to governance and support, experiences of working as or with locums, experiences of being treated by locums, what happens when care goes well or poorly, perceived pros and cons and policies or initiatives to support locums. Data were collected between March 2021 and April 2022.

### 2.3 Analysis

Reflexive thematic analysis (RTA) was used, and a constructionist epistemology was adopted. Implications of this were that, in addition to the recurrence of information in the development of code and themes, meaning and meaningfulness were central criteria in the development of themes.^34^ In RTA, the subjectivity of the method is acknowledged, and the researcher's active role in knowledge generation and subjectivity is regarded as an analytic resource.^35^ In this sense, interpretive variability between researchers based on differences in their knowledge, theoretical assumptions and differences in how they responded to the dataset is expected.^34^ As researchers, we acknowledge that we were not neutral observers but active participants in the construction of knowledge. We were mindful of how our assumptions and experiences shaped our analysis and recognise that themes do not emerge from our data but are constructed through our iterative interpretations and shaped by our positionality and perspectives. Our team has research backgrounds in the health care workforce, professional learning, organisational and professional regulation and quality and safety. Our identities and positionality sensitised us to certain interpretations, including the exclusionary nature of organisational practices, the implications for patient safety and processes of regulation. Drawing on best practices in qualitative research,^36^ we engaged in reflexive dialogue with our team, as well as project advisors and patient and public involvement representatives to foster collaborative meaning making and enhance analytical robustness and the trust worthiness of the data.

In interpreting the data, we reflected critically on the potential for prior knowledge to bias our conclusions. We also considered whether individuals with negative views of locum work were more likely to participate and whether locum doctors felt able to speak openly about negative impacts on their fitness to practice, given potential professional repercussions. To mitigate bias, we sought to represent a diverse range of perspectives, avoiding the privileging of any single viewpoint. Findings were regularly discussed with our project advisory group, which included locum doctors, and with a senior locum who advised on interpretation. These strategies aimed to promote reflexivity and ensure a balanced yet faithful representation of the data.^37^ We determined theoretical sufficiency when we reached a depth of understanding to support a coherent and well‐developed analysis, while recognising that understanding will always be partial, situated and never fully exhaustive.^38^ We triangulated responses from participants from different roles, as well as patients, to provide a more comprehensive understanding of learning and professional development and reduce bias associated with relying on a single data source.^39^, ^40^ Corroborating findings across multiple respondents, not just locums, provides broader insights and a more complete picture of how locum learning is perceived and experienced.

Three members of the research team read and re‐read the transcripts to familiarise themselves with the data; coded the dataset and collated relevant data extracts; generated initial themes and identified broad patterns of meaning; reviewed themes by questioning whether themes answered the research question and told a convincing story of the data combining, splitting and discarding themes as necessary; and defined and named themes by developing a detailed analysis of each theme. Abductive analysis was used and findings were positioned against the extant literature^41^ alongside RTA,^34^ to iteratively engage with both the data and theory. This approach aligns with Braun and Clarke's emphasis on researcher subjectivity, theoretical flexibility and positions the researchers as active interpreters, using theory to guide the analysis and shape interpretation.^34^ However, coding was predominantly inductive, meaning that while coding was theoretically informed by the conceptual frameworks identified in our earlier narrative review and qualitative study,^16^, ^33^ data were open‐coded, and respondent meanings were emphasised. Participant quotes are identified using anonymised codes that indicate their unique identifier and their role (e.g. 35, Locum GP). This coding was designed to preserve anonymity.

## 3 FINDINGS

Findings are presented under four interrelated themes that examine how locum work relates to learning and professional development: (1) exclusion from formal and informal learning opportunities; (2) self‐directed learning and workarounds; (3) decline in knowledge and clinical skills; (4) effects on the professional development of the wider team.

### 3.1 Exclusion from formal and informal learning

The transactional nature of locum contracts meant that locums worked in a way that could disenfranchise them from formal and informal learning processes, which were often organisationally situated. Formal CPD necessary for revalidation was sometimes difficult to access and locums faced challenges in evidencing their learning. Locums were regarded as being contracted predominantly to provide direct clinical care, and their broader professional development or educational needs were not generally regarded as the responsibility of the contracting organisation. This meant that locums could be excluded, or could self‐exclude, from formal and informal educational opportunities.

The trust [hospital] doesn't consider us to have any responsibility for them in regards to development or career progression. (7, Rota Coordinator, Secondary Care)

There will be services that will not even bother to involve the locum 'cause they're like, they're here for three or four weeks. And there will be no feedback given which is also awful because there should be an opportunity to improve, I would want to know if my performance was having a negative impact. (27, Medical Director, Secondary Care)

Locums described professional isolation and being on the periphery of processes and spaces that might enable their development and professional socialisation. For example, one locum described feeling unwelcome in the doctors' lounge and entering this space as a locum as akin to a stranger walking into someone's house. Exclusion from this important social network meant they missed out on relationships with colleagues that would be beneficial for professional development.

The social element is really important and usually neglected, like being told where the mess … doctors' mess is, being allowed into the doctors' mess … but it's more than just a room, for example, it's a community of people. It's a support network. It's much more than that … it would be like someone that you didn't know just walk into your house and make themselves a cup of tea. That's how I would see it. (54, Locum, Secondary Care)

A key reason given for locum exclusion from educational opportunities was that locums were regarded as an expensive resource which meant that organisations did not take on the financial responsibility for providing formal or informal training, which could also include induction and mandatory training. Locums were employed to ‘manage the shop floor’ while permanent staff engaged in training or attended meetings. This meant that locums did not typically have access to CPD, meaning there were substantial differences in the opportunities for professional development and learning for permanent staff in comparison to locums, particularly for short‐term locums.

Never paid for it, never included in it, they don't want to pay you for induction, they are not interested in you coming to any of their meetings and MDTs [multi‐disciplinary teams], and they will certainly not have any kind of CPD engagement with you unless you happened to be there and they happened to have it on already and it didn't make a difference if you were there or not. (35, Locum GP)

If the trust is paying a locum doctor to manage the shop floor, they're not going to be happy for him to be attending a CPD event. So, the way it works is the substantive doctors and the full‐time doctors will actually go and attend a CPD event while the floor would be managed by the locum doctor. You see, so the way that the thing is set up, it does not allow the locum doctor to participate in CPD activities … I've seen through my appraisals the same issues, they are not getting access to CPD. (49, Responsible Officer, Primary and Secondary Care)

However, employing locums only for clinical delivery while excluding them from developmental opportunities was described by some participants as myopic and potentially detrimental for patient safety. There was recognition that organisations should treat locums in the same way as permanent doctors with regard to mandatory training and CPD and that locums were perhaps more in need of training and supervisions because of their peripatetic relationships with colleagues, organisations and governance processes. However, participants described variation in terms of locum inclusion in education and variation in locums wanting to be included. One participant acknowledged that locums could sometimes be ‘abused’ and used to do ‘dirty work’, meaning the clinical delivery they were primarily employed to do may not have been of the same quality as permanent staff, and therefore less likely to lead to professional development.

I think it does depend on the practice and it does depend on the locum. Let's say a practice has got two locums, it might be that one locum is willing [to get involved in CPD] and the other one isn't because it doesn't fit in with whatever else they're doing in the week… Some practices are keen to involve locums, others are not so keen. I think it's just a total melting pot. It's very variable. (59, Locum GP)

The BMA [British Medical Association, professional association and trade union for doctors in the United Kingdom] has a very clear policy on locums. It says that they should be treated the same as other doctors, and they should get CPD. And that makes complete sense for me, to think of locums going round the country. I mean, locums are the people who are in most need of making sure their quality is maintained and they don't have this fixed relationship, and there's no one looking after them. And yet most trusts, or many trusts, will try and take them on and not give them CPD, and just say, oh no, we only want you to do ten clinical sessions. But that's stupid, if you think about it. They get abused, and they often get the worst possible jobs. And to some extent I was guilty of that. I got them to do all the dirty, horrible clinics so that I could concentrate on the management aspect. It's difficult not to do that, as a hard‐pressed consultant, and a locum comes in. (50, Responsible Officer, Secondary Care)

Prioritising the learning of permanent staff and trainees was another key reason for the exclusion of locums from developmental opportunities. Some participants said that they prioritised the development of trainees, partly because opportunities were in short supply but also because they had a responsibility for trainees and a limited time frame in which to train them. Locums described not only missing out to medical trainees but felt they were the ‘bottom of the pile’ if trainees from other professions were vying for opportunities to demonstrate clinical skills.

If I've got a patient who needs a chest drain, you scout about the department and from a training perspective, who am I going to get to come and do it with me. And I will pick an A&E [accident and emergency] trainee over a locum any day, because it's for their training. But factually is that very short sighted because if we're going to develop locum safely, arguably they should have the same training opportunities as the trainees. But when things are in short supply, both the time to train, but also the clinical opportunities, then you do favour the doctors who are on the training programmes. (30, Responsible Officer, Secondary Care)

I tell you what I missed out on like, when all the others would go to training, and because they were trainees, they got these learning opportunities that I didn't get. Say there was a procedure to do that was more complicated, and I'd think, oh, I could do with more experience in that. But, I had to step back. But, even as a trainee that would happen, particularly if you were in, say, a neonatal unit, and they'd got a lot of the trainee ANP, Advanced Nurse Practitioners, you'd end up losing out then. But, as a locum, you lost out even more, because what's the point teaching you to do something, when they've got trainees who'll do it? So, you were bottom of the pile. And, you know, they'd all go off for an afternoon's teaching, and I'd be left on the ward. But, that's my job, that's what I was paid for. (56, Locum, Secondary Care)

### 3.2 Self‐directed learning and workarounds

Given the difficulties accessing development opportunities, locums described having to be proactive and self‐directed in their learning and some had developed a number of workarounds to meet the regulatory clinical governance requirements to produce evidence of CPD and professional development and feedback. These included, working in the same organisations repeatedly and taking on contracts that enabled continuity to facilitate gathering feedback from patients and colleagues as well as involvement in audits. Some locums described seeking out organisations that were perceived as beneficial to learning. Working flexibly meant that locums had more autonomy in how they managed their workload, which for some locums meant more time to dedicate to formal learning.

If you wanted to work towards something to improve your CV [curriculum vitae], if you wanted to do an audit to close the loop three times, which is maximum points for your next application step, you need a bit of consistency to get that done. I mean, I got that done, just from locuming, but I had to sort of break my back, to go out of the way to really make sure that I could get it sorted but it would be much more feasible on a substantive post. (23, Locum, Secondary Care)

I do learn sometimes, it depends, obviously I'm a trainee. I tend to gravitate towards shifts that are more beneficial to my learning than just simple service provision. (54, Locum, Secondary Care)

However, there were concerns about the quality of CPD that locums were accessing. One locum felt that availability and cost were key drivers for locums accessing CPD, rather than the quality of the CPD. Another locum, who had previous experience of employing locums, perceived that locums were more focussed on maximising income than professional development and felt their practice suffered as a consequence. This participant described discontinuing contracts for locums who were not up to date with clinical practice or performing effectively, rather than addressing learning deficits.

Education is a big thing; how do locums get it? Do they get quality education? I think they get whatever is available quickly, often cheaply. They don't get involved quite frequently, certainly not in this area, with the standard background education that all the partners and salaried doctors get. (82, Locum GP)

Locums do work multi‐place because they have to work all the time. They can't just take a lot of time out for education…people who are doing that and making it their full job with a young family they try to maximise their income, and they don't necessarily take time for education. Most fall behind on their education, you're not updating. The locums that we didn't want to continue with would not take very long with the patient, they would not really decide what the problem was correctly, they wouldn't know what to do about modern medical practice, so they were isolated, not up to date with good clinical practice and not treating people well. And that's a terrible thing. (38, Locum Primary and Secondary Care)

### 3.3 Decline in knowledge and clinical skills

Participants described some of the consequences of locum working for learning and professional development. Participants felt that locum working was characterised by a lack of feedback, mentorship and involvement in professional discussions and regular and routine communications, which might subsequently lead to a decline in knowledge and clinical skill. A practice manager in primary care felt that newly qualified doctors working as locums were not ‘fit for purpose’ and presented potential patient safety risks as they were inexperienced doctors practising without feedback, mentorship or the support and supervision of a wider team. A participant who had a permanent position but also worked as a locum felt that locums who were not in training presented higher risks to patients because they may lack experience, knowledge of the local health care system and their skills might not be up to date.

If locuming was a healthy way for new doctors to train then that would be one thing, but it's not; they come out of the scheme very much still needing stabilisers…they just haven't got that peer support, they haven't got that network of going into that practice meeting on a weekly basis going, what do I do? And I've got GPs who have worked here for 20 years, 30 years some of them, and they're still coming in and going, what do I do with this? So why somebody straight of a scheme, never having stood on their own two feet clinically, thinks they have got the measure of what we need a GP – and I mean we as taxpayers, as residents of the UK, not me as a practice manager – they're not fit for purpose. (98, Practice Manager, Primary Care)

I Does the locum typically find out about these complaints or serious untoward incidents?

R Yes, but we put that on the agencies to inform them. I suppose it's not great, really. It's not great for any sort of co‐learning and development but again it comes back to the perception of a locum, you know, they're here just to fill a gap and provide a service and if they're not providing that service then we just remove our responsibility for them. Whereas if that was obviously a doctor that was working here or a substantive doctor we would give them that feedback and put measures in place to make sure that they're getting all the right support and training so that there isn't a repeat of that. I think that's probably the difference that we just see the locums as a service and if they're not providing that service then we just dump it, really. (22, Medical Staffing, Secondary Care)

You don't get that mentorship, you don't get picked up on your mistakes that you would if you were in, you know, a general practice or a clinical meeting. Or every week you'd know what everyone else is doing and you'd be in on the audits that they do to find out when your antibiotics are appropriate, when they're not. I have no idea whether my antibiotic prescribing in hospital is good or not because no one's ever fed it back to me. Whereas if I was a permanent member of staff there, I'd be in that teaching that said our antibiotic prescribing is down and we need to stop prescribing them for upper respiratory tract infections or whatever. So I think that being a locum exacerbates that underlying problem that was there to start with. (53, Locum Secondary Care)

Participants reported that locums were sometimes tasked with different types of work in comparison to permanent staff, which could be described as repetitive, routine or ‘dirty work’. There were concerns that this could lead to deskilling if locums were more likely to be dealing with low risk, acute conditions or minor ailments. One practice manager reported that locums were less likely to gain a breadth of knowledge and experience in general practice, which meant they were less confident than those in permanent roles. Patients were concerned that a lack of patient feedback as a result of discontinuity, combined with funnelling more clinically straightforward cases to locums could lead to a sub‐section of the medical workforce who were not learning enough to practice safety and effectively. However, not everyone agreed that locums were given less difficult work and some locums described being tasked with dealing with more complex and difficult cases.

I think they come straight into this, they're not experienced. They quickly deskill in those chronic long‐term cases because if they get one of those, they just say, oh, you need to come back and see your regular GP next week. So they deal very much with the acute side of the things or the minor ailment side of things because they just do a gap‐fill I suppose for the regular team … they're not gaining that experience and that knowledge of all the different areas of general practice and the different types of diseases. So then they're not confident when it comes to diagnosing a patient and they quickly can pass it on. And it worries me. (3, Practice Manager, Primary Care)

R2: I think that locums are only ever able to scrape the surface and I think that's a shame, and actually because of the nature of their appointments they don't have time to find out enough. And therefore, I think that they should just be dealing with first visits or minor complaints. I do not think they should be standing in for the GPs with people with serious health conditions.

R3: I'd just follow up on that one, are they actually going to learn enough if they're just doing repetitive simple things? (Focus Group C)

### 3.4 Effects on the professional development of the wider team

Locums were frequently employed for service delivery only and not generally obligated or sometimes allowed to supervise clinical trainees, which could have negative consequences for the learning and professional development of the wider team or the ability of the organisation to recruit trainees. Having a locum educational supervisor, who worked sporadic shift patterns or may not return to the organisation, was disruptive and detrimental to the student experience. Not being able to offer training positions meant that organisations, and sometimes whole services, were unable to recruit trainees, which could place an additional burden on existing staff.

In terms of medical students, they tend to get attached to F1s [newly qualified doctors], so they're not meant to be attached to us, but they all started the other day and there wasn't enough of us, so I still do supervise them. But I think one of the other locums said there's not a requirement of us as locums to supervise them, because they're meant to copy our shift pattern, so if I'm only in one or two days a week, then it's not good for the students. (12, Locum, Secondary Care)

One of the things that we struggle with though where we do have long‐term locums is obviously they can't be approved educators, they can't take trainees, or we don't promote it within the trust. So, you kind of end up where whole services are just becoming unable to take trainees. And that is putting more pressure on the people who are substantive. (39, Medical Director, Secondary Care)

And there's consequences of it as well … because as a locum, you can't have a speciality trainee. Which then means we end up in this cycle where we can't attract people to our ward because they can't come and train with us. (89, Psychologist, Secondary Care)

Frequent changes in team composition, due to the transient nature of locum, work meant that locums were not always present for long enough to contribute to the sustained learning or mentorship of colleagues on long‐term development programmes. Inconsistent medical leadership meant disruption to the oversight and support necessary for the learning and development of the wider clinical team.

The locum consultant at the time had said that she'd support me, provide me with the supervision I needed, et cetera, et cetera…And it became quite a mess … And kind of three, four months into it, when, you know, it's a relatively short course, with a lot of work, and the impact of, you know, the stress that I had, because somebody had agreed to support me, but then wasn't there, and then eventually she says, well I'm not coming back now. (87, Nurse, Secondary Care)

## 4 DISCUSSION

Our findings suggest that locum working can be detrimental to the learning and professional development of not only locum doctors, but the wider health care team. The consequences of locum transience and peripheral participation, combined with organisational abdication of responsibility for learning, were weaker relationships with organisations, teams, peers and patients, and exclusion or self‐exclusion from formal and informal learning opportunities. Excluding locums from formal and informal learning opportunities undermines not only doctors' abilities to develop their professional knowledge but also patient safety, team cohesion and fairness. While locum working could offer variety and a breadth of clinical exposure, there were concerns that locum working and organisational approaches to locum inclusion in development opportunities could lead to a degradation, narrowing or stagnation in knowledge and clinical skills, which presented potential risks to patient safety. This is particularly concerning for less experienced junior doctors working as locums, as this period, which is known to be important for the consolidation and crystallisation of knowledge and identity, could be disrupted.^42^

The medical regulator in the UK states that organisations are responsible for creating fair systems and environments that promote learning and enable all doctors to meet their professional obligations and requirements for revalidation.^43^ Despite this responsibility, at a structural level, institutional arrangements and policies constrained equal access to opportunities,^44^ and some organisations did not regard the learning and development of locums as their responsibility, largely due to additional cost and lack of staff capacity. Formal aspects of medical education are different from more general organisational learning, whereby newcomers learn the knowledge and behaviours needed to participate as an effective member of an organisation.^45^ Yet our evidence suggests that locums experienced varying levels of ‘organisational socialisation’^46^ meaning they were not always exposed to the necessary learning and adjustment processes to enable them to function effectively in an organisation.

Locum working, particularly for less experienced doctors, disrupted the ways in which doctors are known to learn in practice.^2^ Research on how doctors learn suggests that with experience comes complex social, behavioural and intuitive wisdom as well as the ability to make comparisons between past and present patients.^47^ Medical education has been described as a process whereby doctors learn to manage uncertainty through pattern recognition and identifying and resolving problems.^2^, ^48^ While some locums reported that locum working accelerated their learning and provided breadth and variety, they also reported professional isolation and lack of feedback. Locum working can disrupt continuity for patients^49^ and transience means that locums may not see the outcomes of their clinical decisions, which could hinder their ability to recognise patterns and make comparisons against similar past patients.^49^, ^50^

Our findings suggest that a lack of organisational integration and involvement in formal and informal development opportunities and feedback meant that locums were often not privy to relevant information that could inform their practice and assist with the identification of areas for focussed development. Disenfranchisement from the teams, mentors and organisational processes meant that locums, and particularly those who worked on a short‐term basis, often had to self‐assess their learning requirements and be proactive, self‐directed learners. Self‐assessment in professional development was once regarded as an individualised, inward‐looking activity, but the importance of the integration of data from external sources has now been recognised.^51^ Evidence suggests doctors demonstrate limited capacity in self‐generated summative assessments of their own performance or ability,^52^ and external assessments and feedback are important in evaluating competence and learning deficits.^53^ Challenges to identifying appropriate and relevant CPD, as well as receiving meaningful feedback are not unique to locums^24^; however, our findings suggest that the temporary nature of locum work, combined with organisational relinquishment of responsibility for the learning and development of locums, exacerbated difficulties in accessing the performance data, mentorship, supervision and colleague and patient feedback necessary for identifying learning deficits.

Medicine operates within a highly structured social framework, where work and learning rely on interaction and are therefore significantly shaped by social power dynamics and hierarchical structures.^54^ We found that a hierarchy of prioritisation was a barrier to locums accessing opportunities, which meant that learning was sometimes perceived to be exclusive to permanent staff and trainees. Competition between medical students for learning opportunities due to ‘overcrowding’ has been reported elsewhere,^55^ and studies of medical education have often focussed on experiences of socialisation and the reproduction of hierarchies,^44^, ^56^ with student doctors often finding themselves at the bottom of these hierarchies.^57^ When it came to accessing learning opportunities, we found that locums could be not only at the bottom of the medical hierarchy but also had to compete with trainees from other professions, such as nursing, which, again, is concerning for more junior doctors who have not yet established technical skills for example.

Clinical placements are integral to medical education and students should be placed in settings that offer the most learning potential.^58^ However, shortages of clinical placements and increases in student numbers, without a corresponding increase in clinical placements, pose significant challenges to expanding the doctor workforce at a time of doctor shortages.^59^ The UK Medical Schools Council called for the number of medical school places to be increased to address the shortage of doctors in the NHS, deal with patient backlogs and achieve greater sustainability.^60^ The Department of Health's consultation proposed expansion of medical school placements as a solution to NHS reliance on ‘expensive locum doctors’.^61^ Our findings indicate that locum working meant some departments, and sometimes whole services, were unable to provide clinical placements for medical trainees, as well as trainees in other professions, because locums were either not permitted to be educators, were unwilling to take on the responsibility, or the length of their contracts or shift patterns meant it was unfeasible. Furthermore, the impact of locum working on medical student placements has not been addressed by NHS England, nor was it mentioned in the Medical School Council's position paper on the expansion of medical student numbers or the Department of Health consultation.^60^, ^61^ Failure to acknowledge how the presence of locum doctors in the NHS workforce affects medical education and the ability to provide placements may hamper the implementation of policies to increase and improve the quality of learning and professional development for the wider health care team.

### 4.1 Strengths and limitations

This large qualitative study explores the implications of locum working for doctors' learning and professional development, an area of medical education and workforce planning that remains under‐researched despite its growing importance. The study focused on sites within England, so findings should be interpreted with caution when thinking about other contexts and systems; similar research in different countries would be valuable to explore whether experiences are replicated elsewhere. It is possible our sample was affected by self‐selection bias and may have included locums, people who worked with locums and patients who had more critical or challenging experiences.

Data collection took place during the COVID‐19 pandemic; collecting data remotely may have narrowed methodological scope as we were unable to carry out observational fieldwork as planned, which could have helped to contextualise interview findings and reduce bias. The pandemic may have also shaped participants' experiences and perspectives, particularly as locum use changed during this period, with fewer locums being deployed and some describing more negative experiences of marginalisation. By combining individual interviews and focus groups, we aimed to accommodate participants' preferences and gather diverse perspectives; we recognise, however, that each method can encourage different types of disclosure and emphasis. Overall, despite these limitations, this study offers important insights into how locum working can affect ongoing learning and professional development and highlights the need for educational strategies and organisational support to help locums engage fully in professional learning across roles and settings.

### 4.2 Future directions for research

While there is some evidence to suggest that locums often experience lower status, weaker integration, and limited support within clinical teams,^16^, ^25^ there remains a significant gap in understanding whether and how racially minoritised doctors face compounded forms of discrimination in locum roles. Future research should explore how locum working intersects with race, ethnicity and other protected characteristics. Investigating this intersectional dimension is critical, particularly given existing evidence of racism and unequal career progression within the wider medical workforce.^62^ Future research should examine how race, gender and migration status shape the locum experience, including perceptions of competence, access to professional development and relationships with permanent staff. Such work would not only deepen our understanding of inequities in temporary medical labour but also inform more inclusive workforce policies.

## 5 CONCLUSION

Our findings indicate that locum working may negatively impact the learning and professional development of both locum doctors and the wider health care team. These findings have important implications for key stakeholders. For professional bodies, the results highlight risks associated with taking on locum work before medical knowledge and experience are established and might persuade doctors to consider working permanently in the early stages of their careers. For organisational leaders, the findings suggest the inclusion of locums in developmental opportunities is important for professional development and quality and safety. For policy makers, our findings highlight the need for a balance between using locums to address the short‐term need to meet workforce quotas and the long‐term impact on the knowledge and development of the workforce and, in turn, patient safety.

## AUTHOR CONTRIBUTIONS

All authors meet the ICMJE criteria for authorship. JF collected and analysed the data and drafted the initial manuscript. All other authors contributed to study design, interpretation of findings and manuscript revisions as well as providing critical input on analysis and theoretical framing. All authors reviewed and approved the final manuscript and agree to be accountable for all aspects of the work.

## ETHICS STATEMENT

The study received ethical approval from the Health Research Authority (IRAS project ID: 278888; REC reference 20:/NW/0386). All participants gave informed consent to be interviewed.

## CONFLICT OF INTEREST STATEMENT

We declare that there are no conflicts of interest in relation to the content, authorship and publication of this manuscript submitted to *Medical Education*.

## Data Availability

Research data are not shared due to privacy or ethical restrictions.
